# Understanding Predictors of Response to Repository Corticotropin Injection Treatment Among Patients With Advanced Symptomatic Sarcoidosis

**DOI:** 10.36469/jheor.2022.33295

**Published:** 2022-04-20

**Authors:** Jas Bindra, Ishveen Chopra, Kyle Hayes, John Niewoehner, Mary P. Panaccio, George J. Wan

**Affiliations:** 1 Falcon Research Group, North Potomac, MD; 2 Manticore Consultancy, Bethesda, MD; 3 Mallinckrodt Pharmaceuticals, Hampton, NJ

**Keywords:** Acthar Gel, repository corticotropin injection, predictors, sarcoidosis, treatment response

## Abstract

**Background:** Sarcoidosis, an inflammatory systemic granulomatous disease, affects multiple organs and has a diverse clinical course. Repository corticotropin injection (RCI) is an effective treatment for advanced symptomatic sarcoidosis. Since sarcoidosis affects patients differently, treatment response may vary by patient demographic, clinical, and treatment-related characteristics and physician specialty. However, there is a paucity of literature regarding predictors of sarcoidosis treatment response.

**Objectives:** This study investigated predictors of response to RCI treatment.

**Methods:** Post-hoc analysis was conducted using data from a previously published retrospective cross-sectional chart review study among symptomatic sarcoidosis patients ≥18 years of age previously treated with RCI. Outcome improvement 3 months post-RCI treatment was based on the clinician’s subjective evaluation and analyzed using adjusted logistic regression. The most influential predictors for each outcome were based on statistical significance (*P*<.05) and the strength of the relationship assessed by the standardized β coefficients.

**Results:** The top predictors of outcome improvements were as follows. **Global health assessment:** (1) improvement in *current health status* influenced by complete RCI compliance, moderate overall symptom severity, and presence of extrapulmonary sites; and (2) improvement in *overall symptoms* influenced by age, shorter duration since sarcoidosis diagnosis, and complete RCI compliance. **Clinical outcomes:** (1) *lung function* improvement influenced by mild weight loss, mild wheezing/coughing, and non–African American race; (2) reduction in *pulmonary fibrosis* influenced by moderate overall symptom severity, mild wheezing/coughing, and mild weight loss; and (3) reduction in *inflammation* influenced by physician specialty, completing a course of RCI treatment, and moderate-to-severe night sweats. **Patient-related outcomes:** (1) reduction in *fatigue* influenced by physician specialty and moderate-to-severe fatigue; and (2) improvement in *quality-of-life* influenced by shorter duration since sarcoidosis diagnosis, moderate-to-severe wheezing/coughing, and complete RCI compliance. *Corticosteroid discontinuation/reduction* was influenced by physician specialty, moderate-to-severe shortness of breath, and comedication use before RCI.

**Conclusions:** RCI may be a better treatment option for patients with more severe disease, primarily those presenting with symptoms. Complete compliance with RCI treatment may improve patients’ health and quality of life. Understanding factors that influence RCI effectiveness across different treatment outcomes in real-world clinical practice is important for designing optimal sarcoidosis treatment strategies.

## INTRODUCTION

Sarcoidosis is an inflammatory systemic granulomatous disease that affects multiple organs and has a diverse clinical course and prognosis.^1^ It has an estimated incidence and prevalence of 8 and 60 cases per 100 000 persons, respectively, in the United States.^2^ It affects the lungs in 90% of patients, and 20%-30% of people with pulmonary sarcoidosis end up with permanent lung damage.^3,4^ Spontaneous remissions may occur in most of the patients; however, 10% to 30% have a chronic progressive disease that can cause permanent organ damage.^1,5^

Sarcoidosis can affect several extrapulmonary organs, including the eyes, heart, joints, liver, lymph nodes, salivary glands, skin, spleen, and nervous system.^6^ The goal of treatment is to improve pulmonary function, maintain organ function, reduce symptoms, prevent organ damage, and improve quality of life (QOL).^3^ Sarcoidosis-associated fatigue has been reported in up to 50%-70% of sarcoidosis patients and is recognized as a disabling symptom, causing impaired QOL.^7^ Prednisone and repository corticotropin injection (RCI; Acthar® Gel) are US Food and Drug Administration–approved therapies for symptomatic sarcoidosis.^8^ Long-term and high-dose corticosteroid use has been linked with increased risk of adverse events and higher health care costs.^9–11^ Chronic exposure to high-dose corticosteroid use nearly doubles the risk of major adverse cardiac events.^12^ Therefore, reduction in corticosteroid use is important for improving a patient’s health. Other off-label treatment options for sarcoidosis comprise antimalarial agents, antimetabolites, immunosuppressants, and tumor necrosis factor inhibitors.^13,14^ However, the evidence supporting the use of these agents is limited.

RCI is used for patients with corticosteroid-refractory disease or intolerance for corticosteroids.^13,15^ Further, the European Respiratory Society recommends the use of RCI as an alternative treatment for patients with intermediate- and high-risk sarcoidosis and continued disease or experiencing a relapse.^16^ RCI is a naturally sourced complex mixture of adrenocorticotropic hormone analogs and other pituitary peptides. As an agonist of all 5 melanocortin receptors, RCI has several potential mechanistic pathways, resulting in the activation of several anti-inflammatory pathways through both glucocorticoid-dependent and glucocorticoid-independent mechanisms that may contribute to its therapeutic effects in sarcoidosis.^17,18^ Studies have shown that RCI is an effective treatment for patients with advanced symptomatic sarcoidosis.^4,19,20^ A retrospective medical record study of 302 patients with symptomatic sarcoidosis suggests that RCI may be a viable treatment option for patients with advanced symptomatic sarcoidosis.^4^ Physician assessment of RCI-treated patients indicated a 95% improvement in overall status and a reduction in corticosteroid use from 61.3% to 12.9% after RCI therapy, with a mean daily dose reduction from 18.2 mg to 9.9 mg.

In light of the findings that RCI is an effective treatment strategy for improving the health status of patients with sarcoidosis, it is important to understand the patient characteristics that may have influenced treatment response. Since sarcoidosis affects patients differently, treatment response may vary by factors such as physician specialty, disease severity, age, ethnicity, and sex. Further, factors predicting improvement may differ by the type of outcome being examined. The previously published medical chart review study of patients with advanced sarcoidosis treated with RCI showed that clinicians used an individualized approach to therapy.^4^ Given the paucity of literature regarding predictors of sarcoidosis treatment response, this study re-analyzed the previously published medical chart review study to better understand patient profiles most likely to benefit from RCI treatment among advanced symptomatic sarcoidosis patients. Insights from this study may help clinicians optimize therapeutic benefits for these difficult-to-treat patients.

## METHODS

### Study Design

This study is a post-hoc analysis using data from a previously published retrospective cross-sectional chart review study. Details on the sample selection and data collection are provided elsewhere.^4^ A representative sample of patients was obtained by merging a national database of RCI prescribers with the American Medical Association Physician Masterfile listing. Physicians from different specialties, including pulmonologists, rheumatologists, primary care physicians, dermatologists, cardiologists, ophthalmologists, gastroenterologists, and neurologists were invited because sarcoidosis may impact multiple organs of the body. Physicians were eligible for this study if they had treated at least 1 patient with symptomatic sarcoidosis with RCI during the previous 36 months.

### Study Sample

The study sample comprised adults 18 years or older with a diagnosis of sarcoidosis who had undergone treatment with RCI in the previous 36 months. Further, patients included had completed an individualized course of RCI (based on the disease severity and initial response of the patient) or had received RCI for at least 6 months at the time of data collection.

### Study Measure

**Response to RCI treatment:** The dependent variables defining outcomes of interest were based on the clinician’s subjective evaluation of the patient’s improvement based on the global assessment (current health status and overall symptoms) after treatment with RCI. Select clinical outcomes (lung function, pulmonary fibrosis, and inflammation), patient-related outcomes (fatigue and patient’s QOL), and treatment-related outcomes (changes in corticosteroid use) were similarly recorded by the clinician. Each of these variables was assessed separately and categorized into two mutually exclusive groups: improvement and no improvement.

Improvement in the patient’s current health status was based on the physician’s response to the question, “What is the patient’s status as of the end of RCI therapy or the 6-month point in therapy for ongoing treatment patients?” Improvement in overall symptoms and individual symptoms was based on the physicians’ response to the question, “Please select the outcomes (overall symptoms, fatigue, lung function, pulmonary fibrosis, inflammation, patient QOL, and corticosteroid use [reduction or discontinuation]) that have improved as a result of RCI treatment.” A patient could have an improvement in more than 1 outcome assessed. These individual treatment responses were selected as they capture the different facets of pulmonary fibrosis.^21,22^ All treatment responses were recorded by the physician in medical charts after RCI initiation in patients with advanced symptomatic sarcoidosis. During the time of assessment, patients had either completed an individualized course of RCI (47%) or had received RCI for at least 6 months (53%) at the time of data collection.

**Predictors of response:** Patient demographic, clinical, and treatment-related variables as well as physician specialty were included to assess the predictors of response (Table 1). All variables were recorded in medical charts by a physician before RCI initiation in patients with advanced symptomatic sarcoidosis. Predictors were selected based on the clinical importance and preliminary descriptive analyses of the data. The categorization was based on the findings from the prior study, preliminary analyses, and sample size.

**Table 1.** Predictors Included in the Unadjusted Logistic Regression Models

**Predictors**	**Definition and Categories**
Patient demographic characteristics	
Age group categories	5 categories: <35 y (reference), 35-44 y, 45-54 y, 55-64 y, and ≥65 y
Sex	Men (reference) and women
Race	Non-African American (reference) and African American
Patient clinical characteristics	
Time since diagnosis (years)	Time since initial sarcoidosis diagnosis
Comorbid conditions	Each comorbid condition was categorized into 2 groups as present or absent (reference). Each comorbid condition was evaluated as a separate variable. These included hypertension, hyperlipidemia, diabetes, heart conditions, respiratory conditions, gastrointestinal conditions, mood disorder, and chronic joint disease/rheumatoid arthritis. Number of comorbidities
Extrapulmonary sites involved	Each extrapulmonary site was categorized into 2 groups as either a site involved or not involved (reference). Each extrapulmonary site was evaluated as a separate variable. These included skin, joints, bone, and liver. Number of extrapulmonary sites involved
Symptoms	Each symptom was categorized into 3 groups as absent (reference), present as mild severity, or present as moderate-to-severe severity. Each symptom was evaluated as a separate variable. These included shortness of breath, fatigue, bone and joint pain, wheezing/coughing, abnormal heartbeats, depressed mood, chest pain, skin rash, anemia, night sweats, weight loss, and eye symptoms.
Patient’s overall symptom severity	3 groups: mild (reference), moderate, severe
Patient treatment characteristics	
No. of comedications used before RCI initiation	Comedication classes considered included biologics, corticosteroids, immunosuppressants, antimalarials, and cytotoxic agents
RCI use	Previous RCI users (reference) and first-time RCI users
Status of RCI treatment at time of data collection	Continuing RCI therapy (reference) and completed a course of RCI therapy
Compliance with RCI treatment	Treatment compliance was assessed at the time of the patient’s visit based on the physician’s response to the question: “On a scale of 1-5, rate the patient’s compliance to Acthar treatment.” The response was measured on a scale of 1 to 5”; where 1 = no compliance, 2-4 = partial compliance, and 5 = complete compliance. No patients reported noncompliance, therefore, only 2 categories, partial (reference) and complete compliance were considered.
RCI initiation dose	≤40 U/wk (reference), 41 U/wk-80 U/wk, and >80 U/wk
Physician characteristics	
Physician specialty assessing patient at time of data collection	4 groups: pulmonologist (reference), rheumatologists, primary care physician, other specialties (dermatologist, cardiologist, ophthalmologist, gastroenterologist, neurologist)

Abbreviation: RCI, repository corticotropin injection.

### Statistical Analyses

Predictors of improvement in treatment response were evaluated using adjusted logistic regression analysis to compute the odds ratio (OR) and 95% confidence interval for each dependent (outcome) variable. The reference category for each response variable was “no improvement.” The OR represents the odds of the occurrence of an outcome given a particular exposure, compared with the odds of the outcome occurring in the absence of that exposure.^23^ First, unadjusted logistic regression analyses were conducted to identify the predictors of each dependent variable, where 1 independent variable was assessed at a time (Table 1). Adjusted logistic regression analyses were conducted by simultaneously including the statistically significant (*P* < 0.05) variables identified in unadjusted logistic regression. The assumption is that these significant variables may influence the outcome, and each of these predictor’s relationships with the dependent variable should be examined by adjusting for other significant predictors in the model. Further, multicollinearity tests were conducted on the variables identified in unadjusted logistic regression to ensure that there was no linear relationship between the independent variables added to the model. Variables with a variance inflation factor value less than 10 or a tolerance value greater than 0.1 were included in the model. In general, the variance inflation factor was less than 2.5.

In addition, the ranking of significant variables in the adjusted model was assessed based on standardized β coefficients. A standardized β coefficient compares the strength of the effect of each independent variable to the treatment response variable relative to other significant independent variables assessed specifically for treatment response.^24^

For all analyses, significance testing was conducted at *a priori* 2-sided β of 0.05. Data were analyzed using Stata version 16.1 (StataCorp, College Station, Texas).

## RESULTS

Characteristics of patients with advanced symptomatic sarcoidosis receiving RCI treatment have been previously published.^4^ From September 2017 to November 2017, eligible physicians provided data on 302 patients with symptomatic sarcoidosis. The study sample had a mean age of 51 years and 52% were women (n = 157). Sixty-four percent of patients (n = 193) had chest imaging and biopsy confirmation of stage 3 or stage 4 sarcoidosis, 30% (n = 90) had been hospitalized for sarcoidosis during the previous 12 months, and 27% (n = 81) had concurrent use of all 3 of the major medication classes: corticosteroids (≥5 mg/day prednisone equivalent for ≥60 days), nonbiological oral medications (eg, hydroxychloroquine, methotrexate, azathioprine, leflunomide, mycophenolate, other nonsteroidal oral agents), and biologics (eg, adalimumab, infliximab, certolizumab, golimumab, etanercept, rituximab).

Based on physician response, the current health status improved by 95%, overall symptoms in 73%, lung function in 38%, inflammation in 33%, QOL in 32%, corticosteroid use reduction or discontinuation in 32%, fatigue in 29%, and pulmonary fibrosis in 11% of the patients.^4^

### Predictors of Treatment Response

Unadjusted logistic regression analysis results are presented in **Supplementary Tables S1 and S2**. Results from the adjusted logistic regression analyses are presented by each treatment response. Statistically significant results based on the adjusted regression model are reported.

### Improvement Based on Global Health Assessment

**Figure 1.**
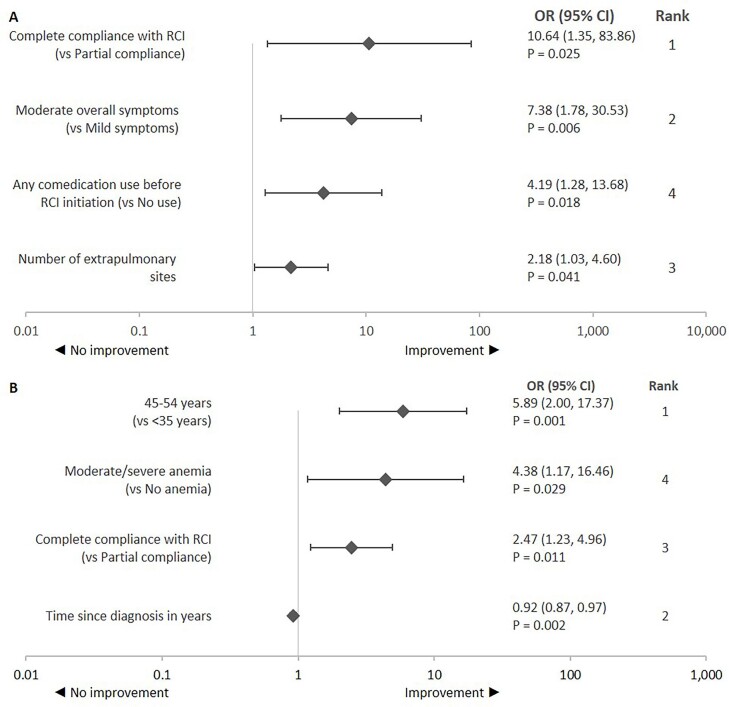
Predictors of Improvement in Global Assessment of Patient’s Health: (**A**) Current Health Status^a^ and (**B**) Overall Symptoms^b^ Abbreviations: CI, confidence interval; OR, odds ratio; RCI, repository corticotropin injection. OR > 1 represents higher odds of improvement and vice versa. Statistical significance was tested at *a priori* β = 0.05. The ranking was assessed based on the standardized β coefficient. Ranks provide relative strength of the relationship between a predictor variable and treatment response relative to other predictor variables that had a significant relationship with the treatment response. ^a^Variables for predicting *improvement in patient’s current health status* included in the adjusted logistic regression model **(A)** were the number of extrapulmonary sites involved, eye symptoms, overall symptom severity, any comedication used 3 months before RCI initiation, and compliance with RCI. ^b^Variables for predicting *improvement in patient’s overall symptoms* included in the adjusted logistic regression model **(B)** were age, time since diagnosis, symptoms (shortness of breath, fatigue, bone and joint pain, anemia, night sweats, weight loss, and eye symptoms), current or prior RCI use, and compliance with RCI.

**Improvement in the patient’s current health status:** Compliance with RCI was the most influential predictor of improvement in the patient’s current health status followed by overall symptom severity and the number of extrapulmonary sites involved (**Figure 1A**). Patients with complete compliance to RCI treatment had significantly higher odds of improvement vs those with partial compliance. Significantly higher odds of improvement in patient’s current health status were also observed with every unit increase in the number of extrapulmonary sites involved, moderate vs mild overall symptom severity, and with any comedication use vs no use 3 months before RCI initiation.

**Improvement in overall symptoms:** Age was the most influential predictor of improvement in the patient’s overall symptoms followed by time since diagnosis and compliance to RCI (**Figure 1B**). Significantly higher odds of improvement were also observed for patients 45 to 54 vs those under 35 years of age and those with moderate-to-severe anemia vs those without anemia. Further, patients with complete compliance with RCI treatment had significantly higher odds of improvement vs those with partial compliance. However, significantly lower odds of improvement were observed with every unit increase in the year of sarcoidosis diagnosis.

### Improvement in Clinical Outcomes

**Figure 2.**
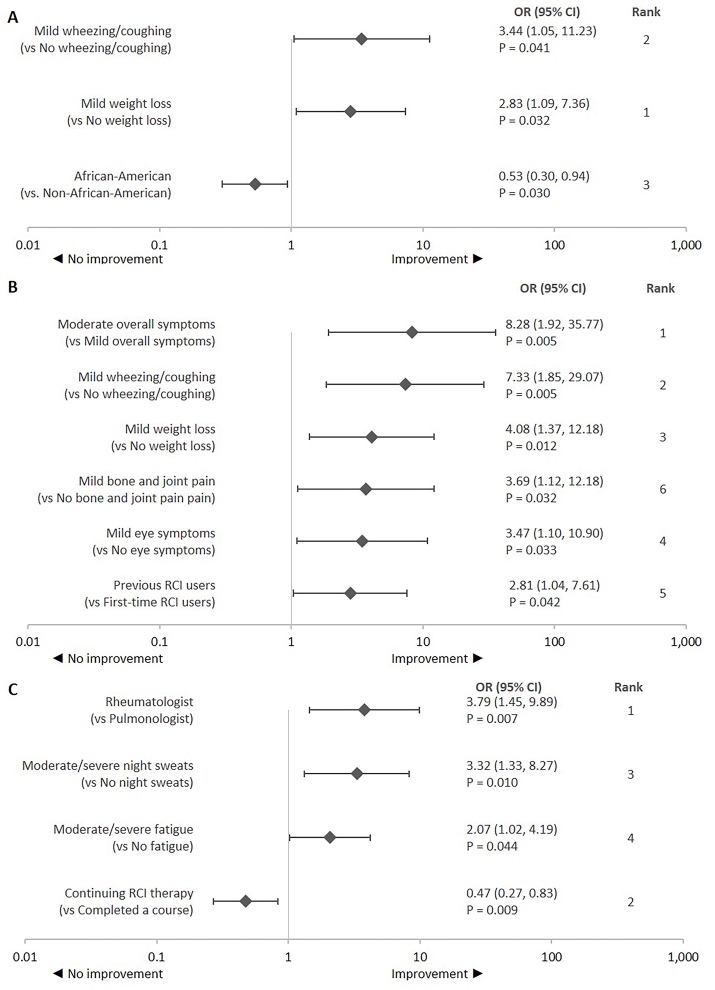
Predictors of Improvement in Clinical Outcomes: (**A**) Lung Function,^a^ (**B**) Pulmonary Fibrosis,^b^ and (**C**) Inflammation^c^ Abbreviations: CI, confidence interval; OR, odds ratio; RCI, repository corticotropin injection. OR > 1 represents higher odds of improvement and vice versa. Statistical significance was tested at *a priori* β = 0.05. The ranking was assessed based on the standardized β coefficient. Ranks provide relative strength of the relationship between a predictor variable and treatment response relative to other predictor variables that had a significant relationship with the treatment response. ^a^Variables for predicting *improvement in lung function* included in the adjusted logistic regression model (**A**) were race, comorbidities (hyperlipidemia, respiratory conditions, gastrointestinal conditions, mood disorder, and chronic joint disease/rheumatoid arthritis), number of comorbid conditions, extrapulmonary sites (skin, joints, and liver), number of extrapulmonary sites involved, symptoms (shortness of breath, bone and joint pain, wheezing/ coughing, chest pain, anemia, night sweats, and weight loss), overall symptom severity, number of comedications used 3 months before RCI initiation, continuing RCI therapy, and initial RCI dose. ^b^Variables for predicting *improvement in pulmonary fibrosis* included in the adjusted logistic regression model (**B**) were race, comorbid respiratory conditions, number of comorbid conditions, symptoms (fatigue, bone and joint pain, wheezing/coughing, night sweats, weight loss, and eye symptom), overall symptom severity, current or prior RCI use, and initial RCI dose. ^c^Variables for predicting *improvement in inflammation* included in the adjusted logistic regression model (**C**) were physician specialty, comorbid conditions (respiratory and gastrointestinal conditions), number of comorbid conditions, extrapulmonary sites (skin, joints, bones, and liver), number of extrapulmonary sites involved, symptoms (fatigue, bone and joint pain, depressed mood, night sweats, and weight loss), overall symptom severity, continuing RCI therapy, and initial RCI dose.

**Improvement in lung function:** Weight loss was the most influential factor for improvement in lung function followed by wheezing or cough symptoms and race (Figure 2A). Significantly higher odds of improvement were observed in patients with mild wheezing or cough and weight loss symptoms vs those without these symptoms. Significantly lower odds of improvement were observed in African Americans vs any other race.

**Reduction in pulmonary fibrosis**: Improvement was defined as a reduction in pulmonary fibrosis. The patient’s overall symptom severity was the most influential predictor of reduction in pulmonary fibrosis followed by wheezing or coughing and weight loss symptom severity (Figure 2B). Significantly higher odds of improvement were observed in patients with mild bone and joint pain, wheezing or coughing, weight loss, and eye symptoms vs those without these symptoms. Significantly higher odds of improvement were also observed for patients with moderate vs mild overall symptoms and previous vs first-time RCI users.

**Reduction in inflammation**: Improvement was defined as a reduction in inflammation. Physician specialty was the most influential factor related to assessment of reduction in inflammation followed by patient’s RCI treatment status and night sweat symptom severity. Rheumatologists had significantly higher odds of reporting improvement in patients than pulmonologists. Patients with moderate-to-severe fatigue or night sweats symptom severity vs those without these symptoms also showed higher odds of improvement. However, significantly lower odds of improvement were observed in those continuing vs those who had completed a course of RCI treatment (Figure 2C).

### Improvement in Patient-Related Outcomes

**Figure 3.**
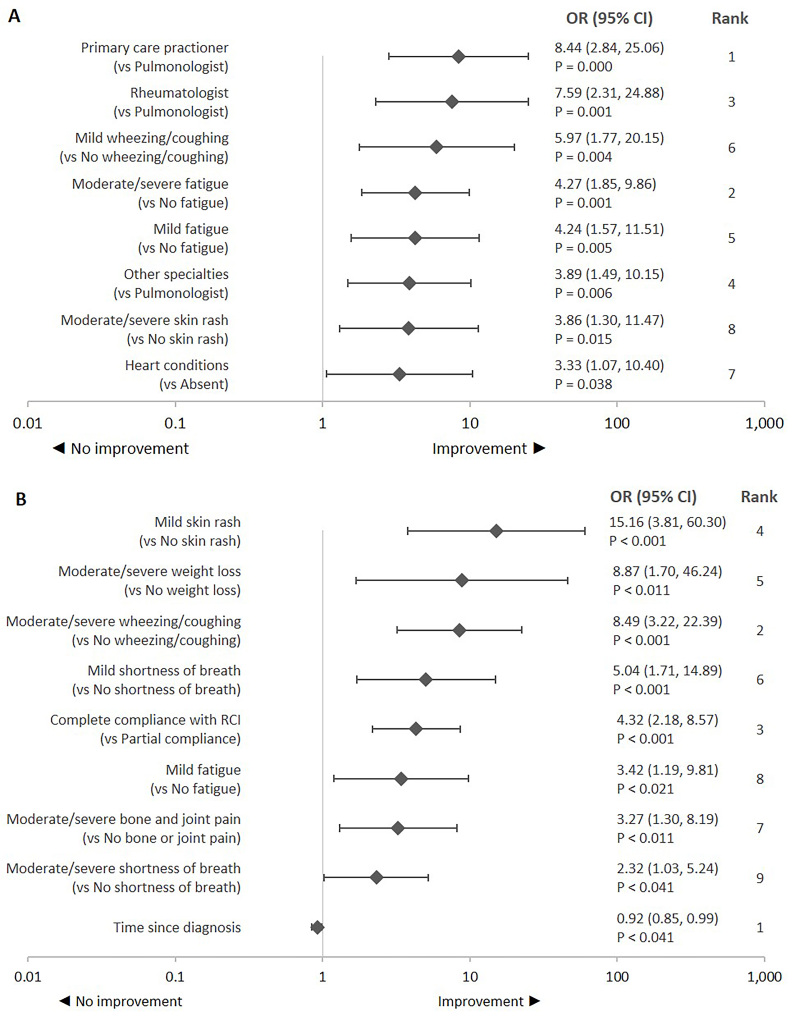
Predictors of Improvement in Patient-Related Outcomes: (**A**) Fatigue^a^ and (**B**) Patient Quality of Life^b^ Abbreviations: CI, confidence interval; OR, odds ratio; RCI, repository corticotropin injection. OR > 1 represents higher odds of improvement and vice versa. Statistical significance was tested at *a priori* β = 0.05. The ranking was assessed based on the standardized β coefficient. Ranks provide relative strength of the relationship between a predictor variable and treatment response relative to other predictor variables that had a significant relationship with the treatment response. ^a^Variables for predicting *improvement in fatigue* included in the adjusted logistic regression model (**A**) were physician specialty, comorbidities (hypertension, hyperlipidemia, heart conditions, respiratory conditions, gastrointestinal conditions, and mood disorder), number of comorbid conditions, extrapulmonary sites (skin, joints, and liver), number of extrapulmonary sites involved, symptoms (shortness of breath, fatigue, bone and joint pain, wheezing/coughing, abnormal heartbeats, depressed mood, chest pain, skin rash, and weight loss), overall symptoms severity, and number of comedications used 3 months before RCI initiation. ^b^Variables for predicting *improvement in quality of life* included in the adjusted logistic regression model (**B**) were time since diagnosis, comorbid respiratory conditions, number of comorbid conditions, extrapulmonary sites (skin and liver), number of extrapulmonary sites involved, symptoms (shortness of breath, fatigue, bone and joint pain, wheezing/coughing, depressed mood, chest pain, skin rash, and weight loss), compliance with RCI, and initial RCI dose.

**Improvement in fatigue**: Improvement was defined as a reduction in fatigue as treatment response. Physician specialty was the most influential predictor of reduction in fatigue (Figure 3A). Rheumatologists, primary care physicians, and other specialties (eg, dermatologist, cardiologist, ophthalmologist, gastroenterologist, neurologist) had significantly higher odds of reporting improvement in fatigue compared with a pulmonologist. Higher odds of improvement were observed in patients with comorbid heart conditions vs those without these comorbid conditions. Higher odds of improvement were also observed in patients with mild or moderate-to-severe fatigue, mild wheezing or coughing, and moderate-to-severe skin rash symptom severity vs those without these symptoms.

**Improvement in patient’s QOL**: Time since diagnosis was the most influential predictor of a patient’s QOL followed by wheezing or coughing symptom severity and compliance with RCI treatment (Figure 3B). Higher odds of improvement were observed in patients with mild or moderate-to-severe shortness of breath, moderate-to-severe bone and joint pain, wheezing or coughing, and weight loss vs those without these symptoms. Further, higher odds of improvement were observed for those with mild fatigue and skin rash symptom severity vs those without these symptoms. Complete vs partial compliance to RCI treatment resulted in significantly higher odds of improvement in QOL. However, significantly lower odds of improvement were observed with every unit increase in time to diagnosis.

### Improvement in Treatment-Related Outcomes

**Figure 4.**
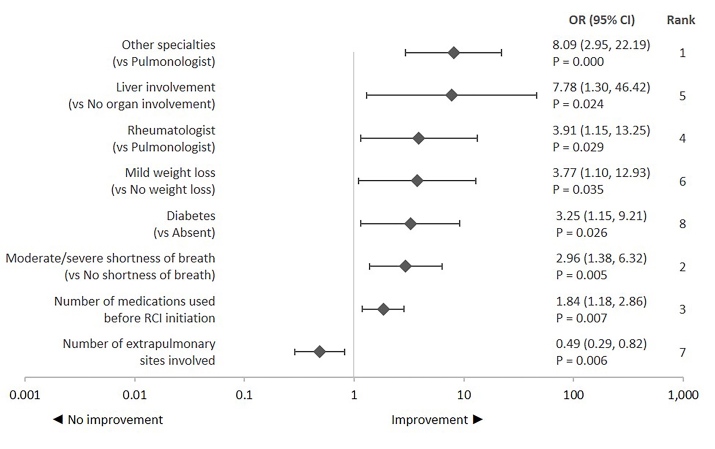
Predictors of Improvement in Treatment-Related Outcomes: Corticosteroid Discontinuation or Reduction Abbreviations: CI, confidence interval; OR, odds ratio; RCI, repository corticotropin injection. OR > 1 represents higher odds of improvement and vice versa. Statistical significance was tested at *a priori* β = 0.05. The ranking was assessed based on the standardized β coefficient. Ranks provide relative strength of the relationship between a predictor variable and treatment response relative to other predictor variables that had a significant relationship with the treatment response. Variables included in the adjusted logistic regression model were physician specialty, comorbidities (hypertension, hyperlipidemia, diabetes, heart conditions, respiratory conditions, gastrointestinal conditions, and chronic joint disease/rheumatoid arthritis), number of comorbid conditions, extrapulmonary sites (skin and liver), number of extrapulmonary sites involved, symptoms (shortness of breath, fatigue, bone and joint pain, wheezing/coughing, depressed mood, chest pain, night sweats, weight loss, and eye symptoms), overall symptom severity, number of comedications used 3 months before RCI initiation, current or prior RCI use, and initial RCI dose. Other specialties: dermatology, cardiology, ophthalmology, gastroenterology, neurology.

Physician specialty was the influential factor related to assessment of corticosteroid discontinuation or reduction followed by shortness of breath symptom severity and comedication use 3 months before RCI initiation (Figure 4). Improvement in treatment-related outcomes was defined as corticosteroid discontinuation or reduction. Rheumatologists or other physician specialties (dermatologist, cardiologist, ophthalmologist, gastroenterologist, neurologist) had higher odds of reporting improvement than a pulmonologist. Among clinical characteristics, higher odds of improvement were observed for patients with comorbid diabetes and those with liver as an extrapulmonary site vs those without these conditions. Further, odds of improvement increased with every unit decrease in the number of extrapulmonary sites involved. Higher odds of improvement in patients with moderate-to-severe shortness of breath or with mild weight loss vs those without this symptom were also observed. Odds of improvement also increased with every unit increase in the number of comedication used 3 months before RCI initiation.

## DISCUSSION

This post-hoc retrospective analysis was conducted to identify predictors of the treatment response with RCI. The top predictors of improvements in outcomes were as follows. *Global health assessment*: (1) improvement in current health status influenced by complete RCI compliance, moderate overall symptom severity, and presence of extrapulmonary sites; (2) improvement in overall symptoms influenced by age, shorter duration since sarcoidosis diagnosis, and complete RCI compliance. *Clinical outcomes*: (1) lung function improvement influenced by mild weight loss, mild wheezing/coughing, and non–African American race; and (2) reduction in pulmonary fibrosis influenced by moderate overall symptom severity, mild wheezing/coughing, and mild weight loss; and (3) reduction in inflammation influenced by physician specialty, completion of a course of RCI treatment, and moderate-to-severe night sweats. *Patient-related outcomes*: (1) reduction in fatigue influenced by physician specialty and moderate-to-severe fatigue; and (2) improvement in QOL influenced by shorter duration since sarcoidosis diagnosis, moderate-to-severe wheezing/coughing, and complete RCI compliance. Corticosteroid discontinuation/reduction influenced by physician specialty, moderate-to-severe shortness of breath, and comedication use before RCI.

**Figure 5.**
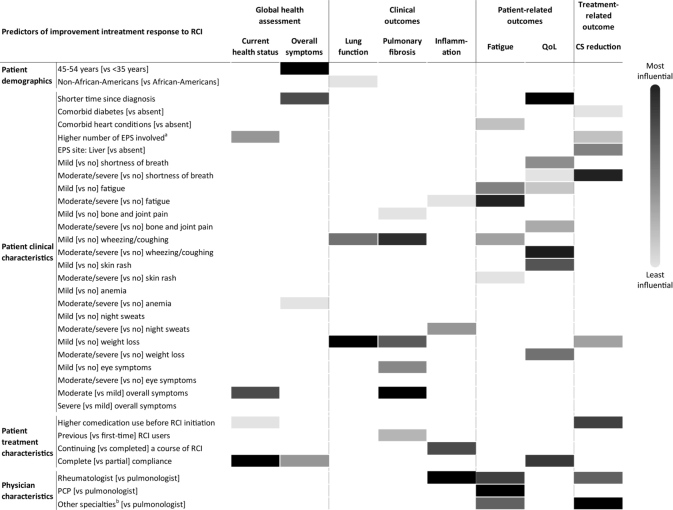
Summary of Predictors of Improvement in Treatment Response to RCI Abbreviations: CS, corticosteroid; EPS, extrapulmonary site; PCP, primary care physician; QOL, quality of life; RCI, repository corticotropin injection. ^a^Improvement in current health status is influenced by a higher number of EPS involved. Reduction in CS use is influenced by a lower number of EPS involved. ^b^Other specialties: dermatology, cardiology, ophthalmology, gastroenterology, neurology.

Clinical factors, primarily symptoms, influenced all outcomes; however, the type and severity of symptom predictors varied with the type of treatment response. Weight loss, shortness of breath, and wheezing or coughing were the most important symptoms that predicted improvement in individual treatment responses. Complete compliance with RCI considerably improved the patient’s current health status, overall symptoms, and QOL compared with partial compliance with RCI. Physician specialty also contributed to differences in assessment of reduction in inflammation, fatigue, and corticosteroid use. Generally, treatment responses did not vary by patient demographics with some exceptions. The findings from the current analysis suggest that patient demographic, clinical, and treatment-level, as well as physician-level variability, exists in predicting outcomes with RCI treatment (Figure 5).

### Improvement Based on Global Health Assessment

Global health assessment is an indicator of improvement in a specific disease or condition. Global health assessments in the current study comprised the patient’s current health status and overall symptoms. Complete compliance with RCI showed considerable improvement in patients’ global health. Patients with complete compliance to RCI showed 10.6 times greater improvement in their current health status and 2.5 times greater improvement in overall symptoms compared with those who were partially compliant. This is consistent with literature that highlights the importance of medication compliance in improving health outcomes among patients with chronic conditions.^25,26^

Patients with greater disease severity may experience a greater benefit with RCI treatment. Improvement in the patient’s current health status with RCI treatment was influenced by moderate overall symptom severity (vs mild disease), a higher number of extrapulmonary site involvement, and any comedication use before RCI initiation. The goal of sarcoidosis treatment is to provide improvement encompassing organ function and symptoms^6^; RCI may be a promising treatment strategy for more “complex” (ie, difficult-to-treat or refractory) patient populations.

Improvement in overall symptoms with RCI treatment was higher in patients in the 45- to 54-year age group than those in the under-35 age group. This difference may be attributed to the greater severity of disease in the older vs younger age group.^27^ Improvement in overall symptoms declined with an increase in the duration of sarcoidosis diagnosis, which may be a function of patient characteristics and disease progression. This is consistent with the literature on other chronic conditions that suggest that the benefits of improvement with treatment are lower for patients with a longer duration of the disease since diagnosis.^28,29^

### Improvement in Clinical Outcomes

Sarcoidosis treatment is aimed at recovering organ function and relieving symptoms.^30^ This study assessed lung function, pulmonary fibrosis, and inflammation as clinical outcomes in patients with sarcoidosis who received RCI. Improvement in clinical outcomes with RCI use was greater in patients presenting with the symptoms; however, improvement in a specific clinical outcome varied with symptom type and severity. Improvement in lung function post-RCI treatment was greater in patients with mild wheezing or coughing and weight loss symptoms than those without these symptoms. Improvement in pulmonary fibrosis was greater in patients with mild wheezing or coughing, weight loss, bone and joint pain, and eye symptoms than those without these symptoms. Improvement in inflammation after RCI initiation was greater among patients with moderate-to-severe fatigue and night sweats symptoms than those without these symptoms. These symptoms are a corollary of the underlying disease and organ damage; these findings highlight the role of RCI in improving organ function.

Improvement in inflammation was also influenced by optimal use of RCI and physician specialty. Patients with a completed course of RCI therapy had higher improvement in inflammation than those who were continuing therapy, suggesting that an optimal course of RCI therapy may be necessary for maximizing benefits. Assessment of improvement in inflammation was related to the physician specialty providing care at the time of data collection; rheumatologists assessed higher improvement compared with pulmonologists. This could be attributed to “inter-physician variation.” Evidence suggests that physicians generally arrive at different diagnostic and treatment decisions for patients albeit with the same clinical symptoms and anamnesis. Sarcoidosis may have features similar to and/or occur concomitantly with several primary rheumatic diseases. Treatment decisions made by physicians for complex diseases, such as sarcoidosis, may lead to variations in outcomes between physicians due to variability in practice style, personal characteristics, and the degree of uncertainty inherent in the practice of medicine. Therefore, rheumatologists’ perspectives may differ from that of pulmonologists’ in assessing improvement in inflammation; pulmonologists may prioritize pulmonary-related clinical outcomes. No difference in the assessment of improvement by physician specialty was observed for patients’ global health, clinical outcomes (lung function and pulmonary fibrosis), and QOL in the current study. This suggests that assessment of improvement in patients’ overall health status and pulmonary function was consistent across all physician specialties. Comprehensive and multidisciplinary care aimed at recovering organ function, relieving symptoms, and improving QOL is usually recommended for patients with sarcoidosis.^30^ Depending on the extent of organ involvement and symptoms, a patient might receive care from a team of different physician specialties.^30^ Further, the diagnostic assessments may change over time with the number of organs involved, new organs being affected, and variability in symptoms.^30^ Because of the variability and heterogeneity of this disease, the patient’s assessment of improvement should be based on the response from all physicians involved in the patient’s care. These findings highlight the need for an integrated care pathway through a multidisciplinary approach to address this complex condition.

Improvement in lung function varied by race. African Americans showed lower improvement in lung function than other races but had a similar rate of improvement (95%) in current health status after RCI treatment compared with the total study population. This study included patients with advanced symptomatic sarcoidosis with lung involvement. Hence, variation in lung function improvement by race may be attributed to the extent of underlying lung damage and more severe pulmonary disease among African Americans. In the United States, African Americans with sarcoidosis experience greater severity of pulmonary disease, a higher extent of multiorgan involvement, worse prognosis, higher rates of hospitalization, and mortality compared with other races.^4,31^ Further, longitudinal studies have shown that African Americans experience the worst sarcoidosis-related outcomes.^32,33^ Together these findings suggest that the degree of sarcoidosis improvement may result from racial disparities inherent to the disease course, driven by an underlying higher severity of the pulmonary disease. As the study did not collect information on lung function parameters (relying solely on the treating physician review of medical records), further clinical assessment is required to better understand this variation.

### Improvement in Patient-Related Outcomes

One of the goals of sarcoidosis treatment is to improve the patient’s QOL. Recent guidance from the European Respiratory Society emphasizes improving patients’ QOL in sarcoidosis.^16^ Our findings showed that improvement in patients’ QOL after RCI use was influenced primarily by the presence of symptoms (type and severity), compliance to RCI, and time since diagnosis. Higher improvement was observed for patients with moderate-to-severe bone and joint pain, wheezing or coughing, and weight loss compared with those without these symptoms. Patients with moderate-to-severe wheezing or coughing had 8.5 times higher odds of improvement in QOL with RCI than those without any wheezing or coughing. Further exploration of data showed that more than three-fourths of patients with moderate-to-severe wheezing or coughing who used RCI for the first time showed improvement. In addition, improvement was higher for patients with either mild or moderate-to-severe shortness-of-breath symptoms. Given that shortness of breath and persistent dry cough are the most common clinical symptoms of sarcoidosis,^34^ these findings suggest that RCI may be a better treatment option for improving QOL in patients with multiple clinical symptoms with greater severity.

Improvement in QOL was also driven by compliance to RCI treatment; patients with complete compliance to RCI showed 4.3 times greater improvement in their QOL compared with those who were partially compliant. The findings from the current analysis are consistent with an observational study on patients with pulmonary sarcoidosis that reported higher medication adherence was associated with better health-related QOL.^35^ These findings have implications for further identifying factors associated with medication adherence to improve clinical and patient-reported outcomes in sarcoidosis. Improvement in fatigue post-RCI use was primarily predicted by mild or moderate-to-severe fatigue, mild wheezing or coughing, and moderate-to-severe skin rash symptoms as well as the presence of comorbid heart conditions before RCI initiation. Improvement in fatigue post-RCI, therefore, indicates improvement in underlying symptoms before RCI initiation. Assessment of improvement in fatigue also varied by physician specialty, with primary care physicians and rheumatologists reporting higher improvement than pulmonologists. This is likely due to inter-physician variation as discussed earlier.

### Improvement in Treatment-Related Outcomes

Evidence suggests that long-term and high-dose corticosteroid use is associated with an increased risk of adverse events and higher health care utilization.^4^ It is important to reduce corticosteroid use to improve patient outcomes. This study examined the predictors of reduction in corticosteroid use after RCI treatment. Improvement was higher among patients with moderate-to-severe shortness of breath vs those without this symptom. Shortness of breath is one of the most common symptoms for patients with sarcoidosis^34^ and may often require corticosteroids for symptom relief. Treatment with RCI can help in improving shortness of breath while reducing the need for corticosteroids and their associated adverse events. Further, after RCI initiation, a greater reduction of corticosteroid use was also observed among patients receiving multiple comedications. Findings from the current study also showed that rheumatologists and other specialties reported a higher reduction in corticosteroid use post-RCI initiation compared with that with pulmonologists. This, too, is likely due to inter-physician variation.

### Study Strengths and Limitations

Sarcoidosis is a heterogeneous disease with multiple extrapulmonary sites involved with varied symptoms.^36^ Management decisions should emphasize a personalized medicine approach based on demographic, clinical, and patient-reported factors to effectively control symptoms, reduce corticosteroid use, and prevent organ damage. To our knowledge, this is the first study to provide insights into factors that predict treatment response to RCI using primary data from one of the largest sarcoidosis case series of patients. This study used ranking analysis (standardized β coefficients) for understanding the importance of predictors in addition to the magnitude and directionality of the relationship between predictors and outcomes. The findings from this study may be generalizable to a larger population of patients with advanced symptomatic sarcoidosis treated with RCI in the United States as probability sampling was used to obtain a nationally representative sample of patients. The current study demonstrated differences in real-world clinical practice and quantifies patient profiles for the achievement of response. The findings add to the nascent literature on improving response to treatments in sarcoidosis and may serve as a blueprint for future studies on understanding the differences contributing to the response to treatments among patients with advanced symptomatic sarcoidosis.

The findings should be interpreted in light of several study limitations. First, this retrospective study utilized data from patient medical records and may have errors and omissions. The impact of potential missing data was minimized by including complete information to the extent possible and relying on information readily available in medical records or best known to the respondents who submitted data for our study. Second, we did not include factors such as diagnostic measurements and safety endpoints that might have limited the clinical predictors included in the study. Third, although physicians’ assessments of improvement in a patient’s health status is a descriptive endpoint to evaluate RCI therapy, there may be a risk of recall and confirmation biases. Further, this subjective endpoint may vary by an individual physician’s interpretation of each patient’s medical record and the physician’s standards for assessing improvement. These may result in overestimation or underestimation of the outcomes with RCI treatment. Fourth, this study did not capture patient perspectives of their global health and QOL outcomes, which may result in underestimation of these outcomes with RCI treatment. A physician’s perception of a patient’s QOL can be very different from a patient’s perspective. Fifth, the data were not collected with the intent for a comparative study, which may also result in selection bias. Sixth, there is a paucity of data on the predictors of response to treatment in patients with sarcoidosis; therefore, it was not possible to explain the interactions between variables. Lastly, the data did not allow for patient differentiation based on their response to other treatments.

## CONCLUSIONS

The findings from the current post-hoc retrospective analysis suggest that patient demographic, clinical, and treatment-level variability, as well as physician-level variability, exist in predicting outcomes with RCI treatment. RCI may be a better treatment option for patients with more severe disease. Weight loss, shortness of breath, and wheezing or coughing were the most important symptoms that predicted improvement in individual treatment responses. Complete compliance with RCI treatment may considerably improve the patient’s health status and QOL. Understanding factors that influence the effectiveness of RCI across different treatment outcomes in real-world clinical practice is important for designing optimal treatment strategies in the management of sarcoidosis.

### Disclosures

This study was sponsored by Mallinckrodt Pharmaceuticals Inc. Repository corticotropin injection (RCI; Acthar® Gel) is a product of Mallinckrodt Pharmaceuticals. KH, JN, and GJW are employees of Mallinckrodt Pharmaceuticals and declare that they have no other conflicts of interest. IC was a research collaborator for the study and declare that they have no conflicts of interest. JB and MPP were paid research consultants for the study and declare that they have no conflicts of interest.

### Contributions

GJW, IC, JB, JN, KH, and MPP were involved in study conceptualization and design. GJW, IC, and JB were involved in study conceptualization and design. GJW, JN, and KH were involved in the statistical analysis design and conducting analysis. GJW, IC, JB, KH, and MPP contributed to the interpretation of the data. GJW, IC, JB, JN, KH, and MPP were involved in the drafting of the manuscript, provided critical review and revision of the manuscript for important intellectual content, and provided the final approval of the version to be published.

## Supplementary Material

Online Supplementary Material
