# Supplementary material for: Understanding Predictors of Response to Repository Corticotropin Injection Treatment Among Patients With Advanced Symptomatic Sarcoidosis
Source: J Health Econ Outcomes Res. 2022 Apr 20;9(1):90–100. doi: 10.36469/jheor.2022.33295 (PMC9021499; doi:10.36469/jheor.2022.33295)
Supplement: Online Supplementary Material [file jheor_2022_9_1_33295_87903.pdf]

Journal of Health Economics and Outcomes Research

Online Supplementary Material

Understanding predictors of response to repository corticotropin injection treatment among patients with advanced symptomatic sarcoidosis. *JHEOR*. 2022;9(1):90-100. doi:10.36469/jheor.2022.33295

Table S1: Unadjusted Logistic Regression Analysis for Predictors of Improvement in Patient's Current Health Status and Overall Symptoms

Table S2: Unadjusted Logistic Regression Analysis for Predictors of Improvement in Individual Treatment Responses

This supplementary material has been provided by the authors to give readers additional information about their work.

This is an open-access article distributed under the terms of the Creative Commons Attribution 4.0 International License (CCBY-4.0). View this license's legal deed at http://creativecommons.org/licenses/by/4.0 and legal code at http://creativecommons.org/licenses/by/4.0/legalcode for more information.

Table S1. Unadjusted Logistic Regression Analysis for Predictors of Improvement in Patient's Current Health Status and Overall Symptoms

**Predictors**	**Treatment Response (Improvement vs No Improvement)**	
	**Current Health Status**[a]	**Overall Symptoms**
Physician characteristics		
Rheumatologist (vs pulmonologist)	4.64 (0.56, 38.29)	1.69 (0.77, 3.75)
Primary care physician (vs pulmonologist)	—	1.24 (0.55, 2.80)
Other specialties[b] (vs pulmonologist)	1.96 (0.62, 6.23)	1.20 (0.66, 2.19)
Demographic characteristics		
35-44 y (vs <35 y)	2.11 (0.28, 15.79)	0.90 (0.37, 2.19)
45-54 y (vs <35 y)	1.21 (0.22, 6.63)	4.33 (1.67, 11.22)**
55-64 y (vs <35 y)	2.71 (0.36, 20.27)	2.22 (0.90, 5.48)
≥65 y (vs <35 y)	0.95 (0.15, 6.10)	1.24 (0.47, 3.26)
Women (vs men)	1.09 (0.37, 3.18)	1.23 (0.74, 2.05)
African Americans (vs other race)	0.94 (0.32, 2.78)	1.01 (0.61, 1.68)
Clinical characteristics		
Time since diagnosis, y	0.99 (0.94, 1.03)	0.93 (0.89, 0.97)**
Number of comorbid conditions	1.29 (0.83, 2.00)	0.99 (0.85, 1.15)
Number of extrapulmonary sites involved	1.98 (1.06, 3.70)*	1.10 (0.88, 1.37)
Comorbid conditions		
Hypertension (vs absent)	2.58 (0.70, 9.47)	1.19 (0.71, 2.01)
Hyperlipidemia (vs absent)	5.44 (0.70, 42.41)	1.63 (0.90, 2.95)
Diabetes (vs absent)	0.39 (0.12, 1.21)	0.84 (0.45, 1.59)
Heart conditions (vs absent)	2.10 (0.27, 16.53)	0.69 (0.34, 1.40)
Respiratory conditions (vs absent)	1.92 (0.24, 15.13)	0.80 (0.38, 1.67)
Gastrointestinal conditions (vs absent)	—	1.80 (0.72, 4.55)
Mood disorder (vs absent)	—	1.34 (0.55, 3.23)
Chronic joint disease/rheumatoid arthritis (vs absent)	1.08 (0.13, 8.64)	0.56 (0.23, 1.35)
Extrapulmonary site involved[c]		
Skin (vs absent)	5.35 (0.69, 41.71)	1.59 (0.88, 2.89)
Joints (vs absent)	4.41 (0.57, 34.45)	1.13 (0.62, 2.05)
Bones (vs absent)	1.57 (0.20, 12.44)	2.19 (0.81, 5.91)
Liver (vs absent)	—	1.98 (0.66, 6.00)
Symptoms		
Shortness of breath (ref: no shortness of breath)		
Mild	2.55 (0.32, 20.54)	2.43 (1.02, 5.83)*
Moderate/severe	1.78 (0.48, 6.66)	1.68 (0.93, 3.04)
Fatigue (ref: no fatigue)		
Mild	—	2.32 (0.97, 5.56)
Moderate/severe	—	1.86 (1.01, 3.41)*
Bone and joint pain (ref: no bone or joint pain)		
Mild	—	4.90 (1.11, 21.52)*
Moderate/severe	—	1.94 (0.97, 3.88)
Wheezing/coughing (ref: no wheezing/coughing)		
Mild	—	1.66 (0.59, 4.63)
Moderate/severe	—	2.04 (0.94, 4.42)
Abnormal heartbeats (ref: no abnormal heartbeats)		
Mild	—	3.33 (0.97, 11.41)

Table S1. Unadjusted Logistic Regression Analysis for Predictors of Improvement in Patient's Current Health Status and Overall Symptoms

Predictors	Treatment Response (Improvement vs No Improvement)	
	Current Health Status[a]	Overall Symptoms
Moderate/severe	—	0.62 (0.30, 1.25)
Depressed mood (ref: no depressed mood)		
Mild	—	1.67 (0.65, 4.26)
Moderate/severe	—	1.61 (0.67, 3.87)
Chest pain (ref: no chest pain)		
Mild	—	1.25 (0.51, 3.07)
Moderate/severe	—	1.29 (0.56, 3.00)
Skin rash (ref: no skin rash)		
Mild	—	1.39 (0.49, 3.91)
Moderate/severe	—	1.63 (0.68, 3.91)
Anemia (ref: no anemia)		
Mild	—	1.20 (0.49, 2.97)
Moderate/severe	—	4.84 (1.11, 21.09)*
Night sweats (ref: no night sweats)		
Mild	—	8.20 (1.08, 62.46)*
Moderate/severe	—	0.96 (0.42, 2.19)
Weight loss (ref: no weight loss)		
Mild	—	6.20 (1.44, 26.73)*
Moderate/severe	—	1.88 (0.61, 5.79)
Eye symptoms (ref: no eye symptoms)		
Mild	0.18 (0.04, 0.73)*	2.43 (0.54, 11.02)
Moderate/severe	1.36 (0.17, 11.04)	0.39 (0.19, 0.81)*
Patient's overall symptom severity (ref: mild symptoms)		
Moderate overall symptom severity	8.26 (2.45, 27.91)**	0.54 (0.27, 1.07)
Severe overall symptom severity	5.07 (0.61, 42.09)	0.59 (0.22, 1.60)
Treatment characteristics		
Number of comedications used before RCI initiation[d]	3.89 (1.23, 12.27)*	0.99 (0.74, 1.32)
Previous RCI users (vs first-time RCI users)	1.51 (0.33, 6.97)	0.48 (0.27, 0.88)*
Continuing RCI therapy (vs completed a course of RCI therapy)	1.13 (0.39, 3.31)	0.82 (0.49, 1.36)
Complete compliance to RCI (vs partial compliance)	9.02 (1.16, 70.15)*	2.20 (1.26, 3.85)**
RCI initiation dose: 41 U/wk to 80 U/wk (vs ≤40 U/wk)	2.90 (0.79, 10.67)	1.25 (0.64, 2.43)
RCI initiation dose: >80 U/wk (vs ≤40 U/wk)	2.68 (0.73, 9.87)	1.08 (0.55, 2.10)

Abbreviation: RCI, repository corticotropin injection.

Results presented as odds ratio (95% confidence interval). Odds ratio >1 represent higher odds of improvement and vice versa. Statistical significance was tested at a priori $\alpha = 0.05$.

Unadjusted logistic regression: ***P < 0.001; **0.001 ≤ P < 0.01; *0.01 ≤ P < 0.05

[a]For the current health status, the sample size was not adequate to analyze all of the selected predictors.

[b]Other specialties: dermatologist, cardiologist, ophthalmologist, gastroenterologist, neurologist.

[c]Only selected extrapulmonary sites have been provided that showed a significant relationship with at least one of the treatment responses in the preliminary analysis.

[d]For current health status outcome, "number of comedications used before RCI initiation" was recategorized into "any comedication use (Yes/No)," due to inadequate sample size.

Table S2. Unadjusted Logistic Regression Analysis for Predictors of Improvement in Individual Treatment Responses

Predictors	Treatment Response (Improvement vs No Improvement)					
	**Lung Function**	**Pulmonary Fibrosis**	**Inflammation**	**Fatigue**	**Quality of Life**	**Corticosteroid Discontinuation or Reduction**
Physician characteristics						
Rheumatologist (vs pulmonologist)	1.17 (0.59, 2.33)	1.20 (0.44, 3.32)	5.46 (2.58, 11.58)***	4.33 (1.93, 9.69)***	1.26 (0.60, 2.66)	2.90 (1.35, 6.26)**
PCP (vs pulmonologist)	0.51 (0.23, 1.17)	1.59 (0.57, 4.45)	2.01 (0.87, 4.64)	6.70 (2.87, 15.65)***	2.01 (0.93, 4.35)	2.16 (0.93, 5.01)
Other specialties[a] (vs pulmonologist)	1.14 (0.65, 1.98)	0.67 (0.26, 1.69)	2.37 (1.25, 4.51)**	2.63 (1.29, 5.37)**	1.44 (0.79, 2.62)	3.43 (1.80, 6.54)***
Demographic characteristics						
35-44 y (vs <35 y)	1.05 (0.42, 2.65)	0.71 (0.18, 2.74)	2.07 (0.83, 5.14)	0.90 (0.34, 2.36)	0.51 (0.20, 1.34)	0.82 (0.30, 2.24)
45-54 y (vs <35 y)	1.27 (0.53, 3.04)	0.46 (0.12, 1.78)	0.77 (0.32, 1.87)	0.71 (0.28, 1.79)	0.82 (0.35, 1.95)	1.67 (0.67, 4.17)
55-64 y (vs <35 y)	1.32 (0.54, 3.20)	1.18 (0.35, 4.01)	0.79 (0.32, 1.95)	1.38 (0.56, 3.42)	0.86 (0.36, 2.08)	1.38 (0.54, 3.51)
≥65 y (vs <35 y)	1.58 (0.60, 4.18)	1.05 (0.27, 4.12)	0.77 (0.28, 2.13)	0.90 (0.32, 2.53)	1.02 (0.39, 2.69)	1.63 (0.59, 4.52)
Women (vs men)	1.54 (0.97, 2.47)	0.80 (0.39, 1.64)	0.94 (0.58, 1.52)	1.60 (0.97, 2.65)	1.29 (0.79, 2.09)	1.45 (0.89, 2.35)
African Americans (vs other races)	0.46 (0.29, 0.74)**	0.39 (0.19, 0.83)*	1.09 (0.67, 1.76)	0.83 (0.50, 1.36)	0.98 (0.60, 1.59)	0.67 (0.41, 1.09)
Clinical characteristics						
Time since diagnosis, y	1.00 (0.96, 1.04)	0.96 (0.87, 1.06)	1.00 (0.96, 1.04)	0.95 (0.89, 1.02)	0.90 (0.84, 0.97)**	1.01 (0.98, 1.05)
Number of comorbid conditions	1.38 (1.20, 1.59)***	1.23 (1.00, 1.52)*	1.27 (1.10, 1.48)**	1.42 (1.21, 1.65)***	1.19 (1.03, 1.38)*	1.62 (1.30, 2.00)***
Number of EPS involved	1.36 (1.09, 1.70)**	1.21 (0.83, 1.79)	1.63 (1.30, 2.05)***	1.47 (1.17, 1.85)**	1.37 (1.11, 1.70)**	1.42 (1.13, 1.77)**
Comorbid conditions						
Hypertension (vs absent)	1.43 (0.89, 2.29)	0.90 (0.43, 1.88)	1.17 (0.72, 1.91)	1.86 (1.13, 3.08)*	1.08 (0.66, 1.77)	2.30 (1.41, 3.77)**
Hyperlipidemia (vs absent)	1.96 (1.18, 3.26)*	0.89 (0.40, 2.00)	0.90 (0.52, 1.53)	1.96 (1.15, 3.34)*	1.22 (0.72, 2.07)	1.92 (1.14, 3.23)*
Diabetes (vs absent)	1.15 (0.63, 2.07)	2.01 (0.90, 4.50)	1.15 (0.63, 2.12)	1.07 (0.57, 2.03)	1.02 (0.55, 1.90)	2.09 (1.16, 3.79)*
Heart conditions (vs absent)	0.91 (0.46, 1.81)	0.37 (0.08, 1.60)	1.19 (0.60, 2.38)	2.40 (1.23, 4.71)*	1.28 (0.64, 2.55)	1.99 (1.02, 3.89)*
Respiratory conditions (vs absent)	4.83 (2.29, 10.19)***	3.57 (1.55, 8.24)**	2.89 (1.45, 5.78)**	2.20 (1.10, 4.41)*	2.43 (1.22, 4.84)*	5.03 (2.44, 10.37)***
Gastrointestinal conditions (vs absent)	3.72 (1.73, 8.02)**	2.41 (0.96, 6.09)	4.21 (1.97, 8.98)***	2.58 (1.23, 5.37)*	1.93 (0.93, 4.03)	2.84 (1.36, 5.91)**
Mood disorder (vs absent)	2.84 (1.32, 6.11)**	2.08 (0.79, 5.52)	2.05 (0.97, 4.35)	2.95 (1.39, 6.28)**	1.90 (0.89, 4.04)	
Chronic joint disease/rheumatoid arthritis (vs absent)	3.30 (1.35, 8.08)**	1.75 (0.56, 5.49)	1.96 (0.83, 4.61)	1.63 (0.68, 3.92)	1.73 (0.73, 4.10)	2.97 (1.25, 7.04)*
EPS involved						
Skin (vs absent)	1.76 (1.06, 2.94)*	1.69 (0.80, 3.55)	2.33 (1.38, 3.91)**	2.01 (1.18, 3.43)*	2.06 (1.22, 3.48)**	1.71 (1.01, 2.89)*
Joints (vs absent)	2.03 (1.19, 3.46)**	1.12 (0.50, 2.53)	1.93 (1.13, 3.32)*	2.00 (1.15, 3.47)*	0.89 (0.49, 1.61)	1.49 (0.86, 2.56)
Bones (vs absent)	1.96 (0.94, 4.10)	1.99 (0.75, 5.27)	2.21 (1.06, 4.64)*	1.53 (0.71, 3.29)	0.97 (0.44, 2.15)	1.28 (0.60, 2.75)
Liver (vs absent)	2.42 (1.03, 5.64)*	2.26 (0.78, 6.52)	5.71 (2.28, 14.29)***	4.68 (1.96, 11.17)**	3.35 (1.43, 7.85)**	7.43 (2.84, 19.42)***

Table S2. Unadjusted Logistic Regression Analysis for Predictors of Improvement in Individual Treatment Responses

**Predictors**	**Treatment Response (Improvement vs No Improvement)**					
	**Lung Function**	**Pulmonary Fibrosis**	**Inflammation**	**Fatigue**	**Quality of Life**	**Corticosteroid Discontinuation or Reduction**
Symptoms						
Shortness of breath (ref: none)						
Mild	1.30 (0.64, 2.62)	1.87 (0.67, 5.21)	1.05 (0.51, 2.16)	1.52 (0.72, 3.20)	2.80 (1.38, 5.66)**	1.88 (0.91, 3.89)
Moderate/severe	2.16 (1.27, 3.65)**	2.09 (0.95, 4.62)	1.13 (0.66, 1.94)	2.30 (1.32, 4.00)**	2.52 (1.45, 4.38)**	3.31 (1.91, 5.73)***
Fatigue (ref: none)						
Mild	1.39 (0.69, 2.81)	2.68 (1.05, 6.87)*	1.75 (0.85, 3.61)	3.31 (1.55, 7.09)**	2.78 (1.36, 5.70)**	0.84 (0.38, 1.85)
Moderate/severe	1.49 (0.88, 2.53)	1.57 (0.69, 3.60)	2.53 (1.47, 4.36)**	6.35 (3.52, 11.46)***	2.69 (1.55, 4.68)***	2.00 (1.16, 3.42)*
Bone and joint pain (ref: none)						
Mild	2.29 (0.96, 5.46)	4.15 (1.44, 11.94)**	2.40 (1.00, 5.74)*	1.65 (0.66, 4.12)	2.84 (1.18, 6.81)*	1.12 (0.44, 2.86)
Moderate/severe	2.55 (1.43, 4.54)**	2.54 (1.12, 5.76)*	2.30 (1.29, 4.11)**	2.39 (1.32, 4.31) **	3.30 (1.83, 5.94)***	2.40 (1.34, 4.28)**
Wheezing/coughing (ref: none)						
Mild	5.56 (2.20, 14.04)***	9.88 (3.63, 26.84)***	1.54 (0.65, 3.63)	5.36 (2.24, 12.85)***	3.63 (1.53, 8.60)**	1.36 (0.55, 3.34)
Moderate/severe	3.27 (1.75, 6.12)***	5.07 (2.15, 11.93)***	1.17 (0.62, 2.23)	4.31 (2.28, 8.15)***	7.95 (4.06, 15.56)***	3.59 (1.91, 6.72)***
Abnormal heartbeats (ref: none)						
Mild	1.28 (0.59, 2.78)	2.09 (0.78, 5.61)	0.85 (0.37, 1.95)	2.43 (1.11, 5.31)*	1.09 (0.48, 2.47)	2.06 (0.94, 4.49)
Moderate/severe	0.70 (0.34, 1.45)	0.43 (0.10, 1.90)	0.65 (0.30, 1.41)	0.78 (0.35, 1.73)	0.72 (0.33, 1.55)	0.87 (0.41, 1.84)
Depressed mood (ref: none)						
Mild	2.02 (0.94, 4.34)	1.78 (0.62, 5.10)	0.97 (0.42, 2.22)	1.42 (0.63, 3.20)	1.20 (0.53, 2.70)	1.74 (0.79, 3.81)
Moderate/severe	1.24 (0.59, 2.58)	1.54 (0.54, 4.35)	2.54 (1.23, 5.27)*	2.52 (1.21, 5.26)*	2.13 (1.03, 4.43)*	3.72 (1.77, 7.81)**
Chest pain (ref: none)						
Mild	1.61 (0.74, 3.53)	1.03 (0.29, 3.68)	0.63 (0.26, 1.55)	0.85 (0.35, 2.09)	1.09 (0.47, 2.51)	0.72 (0.30, 1.77)
Moderate/severe	4.16 (1.93, 8.96)***	2.32 (0.91, 5.91)	1.23 (0.59, 2.59)	2.38 (1.14, 4.95)*	2.41 (1.16, 5.00)*	2.56 (1.23, 5.30)*
Skin rash (ref: none)						
Mild	1.19 (0.49, 2.90)	1.72 (0.54, 5.46)	1.18 (0.47, 2.93)	2.57 (1.06, 6.26)*	5.81 (2.27, 14.91)***	1.96 (0.81, 4.75)
Moderate/severe	1.63 (0.80, 3.32)	0.47 (0.11, 2.07)	1.08 (0.51, 2.27)	3.26 (1.58, 6.74)**	2.03 (0.98, 4.21)	1.77 (0.86, 3.65)
Anemia (ref: none)						
Mild	0.86 (0.37, 1.99)	1.33 (0.43, 4.11)	2.17 (0.97, 4.84)	1.80 (0.80, 4.08)	0.88 (0.37, 2.09)	1.87 (0.83, 4.18)
Moderate/severe	2.58 (1.11, 5.98)*	0.32 (0.04, 2.44)	2.15 (0.94, 4.95)	1.23 (0.51, 3.00)	0.81 (0.33, 2.02)	1.83 (0.79, 4.23)
Night sweats (ref: none)						

Table S2. Unadjusted Logistic Regression Analysis for Predictors of Improvement in Individual Treatment Responses

**Predictors**	**Treatment Response (Improvement vs No Improvement)**					
	**Lung Function**	**Pulmonary Fibrosis**	**Inflammation**	**Fatigue**	**Quality of Life**	**Corticosteroid Discontinuation or Reduction**
Mild	3.91 (1.52, 10.06)**	5.20 (1.90, 14.27)**	2.22 (0.90, 5.45)	1.35 (0.52, 3.48)	1.83 (0.74, 4.53)	1.47 (0.58, 3.70)
Moderate/severe	2.55 (1.18, 5.51)*	2.08 (0.72, 5.99)	3.19 (1.47, 6.91)**	2.06 (0.95, 4.47)	2.13 (0.99, 4.60)	2.73 (1.27, 5.89)*
Weight loss (ref: none)						
Mild	5.99 (2.55, 14.05)***	8.63 (3.60, 20.71)***	2.09 (0.97, 4.51)	5.50 (2.47, 12.22)***	5.15 (2.32, 11.43)***	3.96 (1.81, 8.65)**
Moderate/severe	5.44 (2.03, 14.58)**	3.05 (0.92, 10.03)	3.19 (1.29, 7.90)*	2.39 (0.96, 5.95)	5.97 (2.30, 15.47)***	2.90 (1.18, 7.15)*
Eye symptoms (ref: none)						
Mild	0.71 (0.24, 2.12)	3.95 (1.27, 12.28)*	2.32 (0.84, 6.41)	0.14 (0.02, 1.11)	1.05 (0.35, 3.14)	1.05 (0.35, 3.14)
Moderate/severe	0.97 (0.47, 2.03)	0.84 (0.24, 2.95)	2.06 (0.99, 4.26)	0.56 (0.23, 1.34)	1.83 (0.88, 3.79)	2.32 (1.12, 4.78)*
Patient's overall symptom severity (ref: mild symptoms)						
Moderate overall symptom severity	2.41 (1.25, 4.64)**	5.08 (1.17, 21.96)*	2.06 (1.05, 4.05)*	1.61 (0.82, 3.18)	0.90 (0.49, 1.66)	2.60 (1.25, 5.43)*
Severe overall symptom severity	5.22 (2.07, 13.14)***	3.21 (0.51, 20.31)	3.92 (1.56, 9.88)**	3.46 (1.37, 8.73)**	2.20 (0.92, 5.27)	10.31 (3.81, 27.89)***
Treatment characteristics						
Number of comedications used before RCI initiation	1.35 (1.03, 1.76)*	0.74 (0.49, 1.12)	1.26 (0.95, 1.67)	1.41 (1.06, 1.88)*	1.25 (0.95, 1.64)	2.04 (1.51, 2.75)***
Previous RCI users (vs first-time RCI users)	1.41 (0.79, 2.49)	2.91 (1.36, 6.23)**	1.74 (0.97, 3.10)	1.05 (0.57, 1.95)	0.74 (0.39, 1.39)	1.97 (1.10, 3.52)*
Continuing RCI therapy (vs completed a course of RCI therapy)	0.47 (0.29, 0.75) **	0.58 (0.28, 1.21)	0.40 (0.25, 0.66)***	0.96 (0.58, 1.58)	0.62 (0.38, 1.01)	0.95 (0.58, 1.53)
Complete compliance to RCI (vs Partial compliance)	1.14 (0.71, 1.84)	1.86 (0.91, 3.82)	0.66 (0.40, 1.10)	1.25 (0.75, 2.07)	2.79 (1.69, 4.60)***	1.59 (0.97, 2.60)
RCI initiation dose: 41 U/wk to 80 U/wk (vs ≤40 U/wk)	2.25 (1.14, 4.44)*	2.11 (0.57, 7.88)	1.28 (0.65, 2.55)	1.14 (0.58, 2.24)	1.33 (0.67, 2.65)	1.74 (0.85, 3.58)
RCI initiation dose: >80 U/wk (vs ≤40 U/wk)	3.06 (1.54, 6.07)**	4.59 (1.31, 16.12)*	2.44 (1.24, 4.79)*	1.43 (0.73, 2.82)	2.03 (1.03, 4.00)*	3.18 (1.56, 6.48)**

Abbreviations: EPS, extrapulmonary site; PCP, primary care physician; RCI, repository corticotropin injection.

Results presented as odds ratio (95% confidence interval). Odds ratio >1 represent higher odds of improvement and vice versa. Statistical significance was tested at a priori β = 0.05.

Unadjusted logistic regression: ***$P < 0.001$; **$0.001 \le P < 0.01$; *$0.01 \le P < 0.05$.

[a]Other specialties: dermatologist, cardiologist, ophthalmologist, gastroenterologist, neurologist.

[b]Only selected extrapulmonary sites have been provided that showed a significant relationship with at least 1 of the treatment responses in the preliminary analysis.
